# The comorbidity and co-medication profile of patients with progressive supranuclear palsy

**DOI:** 10.1007/s00415-023-12006-4

**Published:** 2023-10-06

**Authors:** Stephan Greten, Florian Wegner, Ida Jensen, Lea Krey, Sophia Rogozinski, Meret Fehring, Johanne Heine, Johanna Doll-Lee, Monika Pötter-Nerger, Molly Zeitzschel, Keno Hagena, David J. Pedrosa, Carsten Eggers, Katrin Bürk, Claudia Trenkwalder, Inga Claus, Tobias Warnecke, Patrick Süß, Jürgen Winkler, Doreen Gruber, Florin Gandor, Daniela Berg, Steffen Paschen, Joseph Classen, Elmar H. Pinkhardt, Jan Kassubek, Wolfgang H. Jost, Lars Tönges, Andrea A. Kühn, Johannes Schwarz, Oliver Peters, Eman Dashti, Josef Priller, Eike J. Spruth, Patricia Krause, Annika Spottke, Anja Schneider, Aline Beyle, Okka Kimmich, Markus Donix, Robert Haussmann, Moritz Brandt, Elisabeth Dinter, Jens Wiltfang, Björn H. Schott, Inga Zerr, Mathias Bähr, Katharina Buerger, Daniel Janowitz, Robert Perneczky, Boris-Stephan Rauchmann, Endy Weidinger, Johannes Levin, Sabrina Katzdobler, Emrah Düzel, Wenzel Glanz, Stefan Teipel, Ingo Kilimann, Johannes Prudlo, Thomas Gasser, Kathrin Brockmann, Daniel C. Hoffmann, Thomas Klockgether, Olaf Krause, Johannes Heck, Günter U. Höglinger, Martin Klietz

**Affiliations:** 1 Department of Neurology, Hannover Medical School, Carl-Neuberg-Straße 1, 30625 Hannover, Germany; 2 Department of Neurology, University Medical Center Hamburg-Eppendorf, Martinistr. 52, 20246 Hamburg, Germany; 3 Department of Neurology, University Hospital of Marburg and Gießen, 35043 BaldingerstraßeMarburg, Germany; 4 Department of Neurology, Knappschaftskrankenhaus Bottrop, Osterfelder Str. 157, 46242 Bottrop, Germany; 5 Kliniken Schmieder Stuttgart-Gerlingen, Solitudestraße 20, 70839 Gerlingen, Germany; 6 Paracelsus-Elena Klinik, Klinikstraße 16, 34128 Kassel, Germany; 7 Department of Neurology with Institute of Translational Neurology, University Hospital Muenster, Albert-Schweitzer-Campus 1, 48149 Muenster, Germany; 8 Department of Neurology and Neurorehabilitation, Klinikum Osnabrueck-Academic Teaching Hospital of the WWU Muenster, Am Finkenhügel 1, 49076 Osnabrueck, Germany; 9 Department of Molecular Neurology, University Hospital Erlangen, Friedrich-Alexander-Universität Erlangen-Nürnberg, Schloßplatz 4, 91054 Erlangen, Germany; 10 Center of Rare Diseases Erlangen (ZSEER), University Hospital Erlangen, Friedrich-Alexander-Universität Erlangen-Nürnberg, Schloßplatz 4, 91054 Erlangen, Germany; 11 Movement Disorders Hospital, Beelitz-Heilstätten, Straße Nach Fichtenwalde 16, 14547 Beelitz-Heilstätten, Germany; 12 Department of Neurology, Kiel University, Christian-Albrechts-Platz 4, 24118 Kiel, Germany; 13 Department of Neurology, University of Leipzig Medical Center, Liebigstraße, 18, 04103 Leipzig, Germany; 14 Department of Neurology, University of Ulm, Oberer Eselsberg 45, 89081 Ulm, Germany; 15 German Center for Neurodegenerative Diseases (DZNE), Oberer Eselsberg, 89081 Ulm, Germany; 16 Parkinson-Klinik Ortenau, Kreuzbergstraße 12, 77709 Wolfach, Germany; 17 Department of Neurology, St. Josef-Hospital, Ruhr University Bochum, Gudrunstraße 56, 44791 Bochum, Germany; 18 Protein Research Unit Ruhr (PURE), Neurodegeneration Research, Ruhr University Bochum, Universitätsstraße 150, 44801 Bochum, Germany; 19 Movement Disorder and Neuromodulation Unit, Department of Neurology, Charité, University Medicine Berlin, Charitépl. 1, 10117 Berlin, Germany; 20 German Center for Neurodegenerative Diseases (DZNE), Charitépl. 1, 10117 Berlin, Germany; 21 Department of Neurology, Klinik Haag I. OB, Krankenhausstraße 1, 84453 Mühldorf a. Inn, Germany; 22 Department of Psychiatry, Charité-Universitätsmedizin Berlin, Charitépl. 1, 10117 Berlin, Germany; 23 Department of Neurology, Charité-Universitätsmedizin Berlin, Charitépl. 1, 10117 Berlin, Germany; 24 Department of Psychiatry and Psychotherapy, Charité, Charitépl. 1, 10117 Berlin, Germany; 25 Department of Psychiatry and Psychotherapy, Klinikum Rechts der Isar, Technical University Munich, Ismaninger Str. 22, 81675 Munich, Germany; 26 German Center for Neurodegenerative Diseases (DZNE), Venusberg-Campus 1, 53127 Bonn, Germany; 27 Department of Neurology, University Hospital Bonn, Venusberg-Campus 1, 53127 Bonn, Germany; 28 Department of Neurodegenerative Diseases and Geriatric Psychiatry, University Hospital Bonn, Venusberg-Campus 1, 53127 Bonn, Germany; 29 German Center for Neurodegenerative Diseases (DZNE), Tatzberg 41, 01307 Dresden, Germany; 30 Department of Psychiatry and Psychotherapy, University Hospital Carl Gustav Carus, Technische Universität Dresden, Fetscherstraße 74, 01307 Dresden, Germany; 31 Department of Neurology, University Hospital Carl Gustav Carus, Technische Universität Dresden, Fetscherstraße 74, 01307 Dresden, Germany; 32 German Center for Neurodegenerative Diseases (DZNE), Von-Siebold-Str. 3a, 37075 Göttingen, Germany; 33 Department of Psychiatry and Psychotherapy, University Medical Center Goettingen, University of Göttingen, Von-Siebold-Str. 5, 37075 Göttingen, Germany; 34 Neurosciences and Signaling Group, Institute of Biomedicine (iBiMED), Department of Medical Sciences, University of Aveiro, Campus Universitário de Santiago, 3810-193 Aveiro, Portugal; 35 Department of Neurology, University Medical Center, Georg August University, Von-Siebold-Str. 5, 37075 Göttingen, Germany; 36 Cluster of Excellence Nanoscale Microscopy and Molecular Physiology of the Brain (CNMPB), University Medical Center Göttingen, Von-Siebold-Str. 5, 37075 Göttingen, Germany; 37 German Center for Neurodegenerative Diseases (DZNE), Feodor-Lynen-Strasse 17, 81377 Munich, Germany; 38 Institute for Stroke and Dementia Research, University Hospital, LMU Munich, Feodor-Lynen-Strasse 17, 81377 Munich, Germany; 39 Department of Psychiatry and Psychotherapy, University Hospital, LMU Munich, Feodor-Lynen-Strasse 17, 81377 Munich, Germany; 40 Munich Cluster for Systems Neurology (SyNergy) Munich, Feodor-Lynen-Strasse 17, 81377 Munich, Germany; 41 Ageing Epidemiology Research Unit, School of Public Health, Imperial College London, Exhibition Rd, South Kensington, London, SW7 2BX UK; 42 Department of Neurology, University Hospital of Munich, Ludwig-Maximilians-Universität (LMU) Munich, Feodor-Lynen-Strasse 17, 81377 Munich, Germany; 43 German Center for Neurodegenerative Diseases (DZNE), Leipziger Straße 44, 39120 Magdeburg, Germany; 44 Institute of Cognitive Neurology and Dementia Research, Otto-von-Guericke University, Universitätspl. 2, 39106 Magdeburg, Germany; 45 Institute of Cognitive Neuroscience, University College London, Gower St, London, WC1E 6BT UK; 46 Clinic for Neurology, Medical Faculty, University Hospital Magdeburg, Leipziger Str. 44, 39120 Magdeburg, Germany; 47 German Center for Neurodegenerative Diseases (DZNE), Gehlsheimer Straße 20, 18147 Rostock-GreifswaldRostock, Germany; 48 Department of Psychosomatic Medicine, Rostock University Medical Center, Schillingallee 35, 18057 Rostock, Germany; 49 Department of Neurology, University Medical Center, Schillingallee 35, 18057 Rostock, Germany; 50 German Center for Neurodegenerative Diseases (DZNE), Otfried-Müller-Straße 23, 72076 Tübingen, Germany; 51 Department of Neurodegenerative Diseases, Hertie Institute for Clinical Brain Research, University of Tübingen, Hoppe-Seyler-Straße 3, 72076 Tübingen, Germany; 52 Center for Medicine of the Elderly, DIAKOVERE Henriettenstift and Department of General Medicine and Palliative Care, Hannover Medical School, Carl-Neuberg-Straße 1, 30625 Hannover, Germany; 53 Center for Geriatric Medicine, Hospital DIAKOVERE Henriettenstift, Schwemannstrasse 19, 30559 Hannover, Germany; 54 Institute for Clinical Pharmacology, Hannover Medical School, Carl-Neuberg-Straße 1, 30625 Hannover, Germany

**Keywords:** Progressive supranuclear palsy, Comorbidities, Polypharmacy, Drug–drug interactions

## Abstract

**Background:**

Progressive supranuclear palsy (PSP) is usually diagnosed in elderly. Currently, little is known about comorbidities and the co-medication in these patients.

**Objectives:**

To explore the pattern of comorbidities and co-medication in PSP patients according to the known different phenotypes and in comparison with patients without neurodegenerative disease.

**Methods:**

Cross-sectional data of PSP and patients without neurodegenerative diseases (non-ND) were collected from three German multicenter observational studies (DescribePSP, ProPSP and DANCER). The prevalence of comorbidities according to WHO ICD-10 classification and the prevalence of drugs administered according to WHO ATC system were analyzed. Potential drug–drug interactions were evaluated using AiD*Klinik*®.

**Results:**

In total, 335 PSP and 275 non-ND patients were included in this analysis. The prevalence of diseases of the circulatory and the nervous system was higher in PSP at first level of ICD-10. Dorsopathies, diabetes mellitus, other nutritional deficiencies and polyneuropathies were more frequent in PSP at second level of ICD-10. In particular, the summed prevalence of cardiovascular and cerebrovascular diseases was higher in PSP patients. More drugs were administered in the PSP group leading to a greater percentage of patients with polypharmacy. Accordingly, the prevalence of potential drug–drug interactions was higher in PSP patients, especially severe and moderate interactions.

**Conclusions:**

PSP patients possess a characteristic profile of comorbidities, particularly diabetes and cardiovascular diseases. The eminent burden of comorbidities and resulting polypharmacy should be carefully considered when treating PSP patients.

## Introduction

Progressive supranuclear palsy (PSP) is a rare neurodegenerative movement disorder with a mean onset between 60 and 66 years of age [1, 2]. Hence, PSP is a disease of elderly people, who often suffer from various additional chronic diseases. A previous report detected diabetes mellitus and cerebrovascular diseases as characteristic pre-diagnostic accompanying disorders of PSP.[3] A similar pattern of comorbidities in PSP, including arterial hypertension, was found in two other cohorts from Western countries [4, 5].

Treatment of the diverse motor and non-motor symptoms of PSP often requires the administration of multiple drugs [6, 7]. Together with the drug therapy necessary for comorbidities, this can result in polypharmacy [8]. The amount of administered drugs and therefore the prevalence of polypharmacy increases dramatically with patients’ age up to > 40% in people aged 85 years or older [9, 10]. Polypharmacy is a leading cause for drug-related problems, e.g., adverse drug reactions (ADR) or drug–drug interactions (DDIs), that may result in falls, hospitalizations or death [11–13].

Since patients with PSP represent a highly vulnerable group, multimorbidity and polypharmacy should be given special attention in medical care. There currently is a lack of detailed knowledge on comorbidities and specific aspects of drug therapy in patients with PSP. In this study, we aimed to analyze the comorbidity profile and particular issues of drug therapy in PSP patients from two large German, multicenter PSP cohorts compared to a German, multicenter cohort of patients without neurodegenerative diseases. In addition, we elaborated aspects of drug safety in PSP patients with different disease phenotypes.

## Methods

### Participants

Ethical approvals were obtained from the local Ethics Committees of all participating study centers. The data analysis of the study was additionally amended to the Ethics Committee at Hannover Medical School (No. 3558–2017, amendment in 2020). Cross-sectional data of 350 PSP patients were collected within two German, multicenter, observational cohort studies, the ProPSP study (German Parkinson and Movement Disorders Society, DPG) and the DescribePSP study (German Center for Neurodegenerative Diseases, DZNE) [14, 15]. PSP diagnosis and phenotype were determined by expert neurologists according to Movement Disorders Society diagnostic criteria for PSP (MDS-PSP criteria) [16]. In case of multiple allocations of phenotypes to one patient, the clinical phenotype was defined using the Multiple Allocations eXtinction (MAX) rules [17]. PSP with predominant Parkinsonism (PSP-P), with predominant corticobasal syndrome (PSP-CBS), with predominant progressive gait freezing (PSP-PGF), with predominant frontal presentation (PSP-F), with predominant ocular motor dysfunction (PSP-OM), with predominant postural instability (PSP-PI) and with predominant speech/language disorder (PSP-SL), were summarized as variant phenotypes (vPSP). The data of 363 patients from multicenter cohort study DANCER (DZNE) were used for a comparison (non-ND). Relatives of patients with neurological diseases, interested persons and neurological patients without neurodegenerative disease were participating in this study. As the DANCER cohort accordingly included young patients (lowest age: 20 years), the comparability with the PSP cohort was established by selection for age. Therefore, the age of selection was increased until no significant difference persisted in the age distribution of the PSP and non-ND group. This cut-off point was ≥ 57 years of age. In this way, the data of 335 PSP and 275 non-ND patients could be compared. Participants did not receive any financial compensation for participating in the study.

### Data acquisition

Experienced movement disorder specialists in all participating centers together with study nurses performed the survey and examination. Demographic information (age, sex and symptom onset), clinical scales (CGI, Clinical Global Impression; MoCA, Montreal Cognitive Assessment; PSPSS, Progressive Supranuclear Palsy Staging System; PSP-RS, Progressive Supranuclear Palsy Rating Scale; GDS-15, Geriatric Depression Scale-15) and medical history (comorbidities and medication) were collected from patients or their caregivers, if a proper survey could not be performed. Data from the most recent visit were used for analysis. The comorbidities were classified according to the first and second level of the World Health Organization (WHO) International Classification of Diseases, 10th Revision (WHO ICD-10, latest version, 2019). Only ongoing conditions or diseases that required regular medical check-up or continuous treatment were included. The medication was classified according to the WHO Anatomical Therapeutic Chemical (ATC) system. The levodopa equivalent dose (LED) was calculated as described previously [18]. Potential drug–drug interactions (pDDIs) were identified using the clinical decision support system (CDSS) AiD*Klinik*® (AID, version 01.05.2020; Dosing GmbH, Heidelberg, Germany) [19–21]. The analysis did not consider whether pDDIs resulted in actual side effects. PDDIs were differentiated according to their severity ranging from “disputed evidence,” “light interaction,” “moderate interaction,” and “severe interaction” to “contraindicated combination.” Patients aged ≥ 70 years, with multimorbidity (≥ three ongoing diseases) and polypharmacy (≥ five long-term drugs) were defined as “geriatric.”[22]

## Statistical analysis

Descriptive statistical analyses were performed using GraphPad Prism 9 (GraphPad Prism Software Inc., San Diego, California) and IBM SPSS Statistics 27 **(**IBM, Corp., Armonk, New York, USA). Continuous variables are reported as mean and standard deviation (SD). To test for normal distribution, the Shapiro–Wilk and Kolmogorov–Smirnov tests were used. In case of a normal distribution, the unpaired t test was carried out to detect significant differences; in case of non-normal distribution, the Mann–Whitney U test was used. Chi-squared test was performed to compare proportions for categorical variables (e.g., sex, prevalence). Odds ratios (OR) are displayed together with the 95% confidence intervals.

## Results

### Patient characteristics

All demographic characteristics are shown in Table 1. PSP (*n* = 335) and non-ND (*n* = 275) were similar in age (PSP, 71.1 ± 6.7 years; non-ND, 70.0 ± 7.1 years; *p* = 0.090) but offered a different sex distribution (PSP, 151 (45.1% females); non-ND, 146 (53.1% females); *p* = 0.049). The PSP group met the characteristics (age ≥ 70 years, multimorbidity and polypharmacy) of geriatric patients significantly more often (PSP, 97 (29.0%); non-ND, 20 (7.3%); *p* < 0.001).

**Table 1:** Main demographic and clinical characteristics of PSP and non-ND patients

Characteristic	PSP (*n* = 335)	Non-ND (*n* = 275)
Age, mean ± SD (min, max)	71.1 ± 6,7 (57, 88)	70.0 ± 7.1 (57, 91)
Sex, female (%)	151 (45.1)	146 (53.1)*
Geriatric patients, *n* (%)	98 (29.2)	20 (7.2)***
Age ≥ 70, *n* (%)	193 (57.6)	148 (53.8)
Multimorbidity, *n* (%)	212 (63.3)	163 (59.3)
Polypharmacy, *n* (%)	181 (54.0)	56 (20.4)***

Abbreviations: *PSP* progressive supranuclear palsy; *SD* standard deviation

^*^p < 0.05, **p < 0.01, ***p < 0.001, Chi-squared test

### Comorbidities

**Fig. 1:**
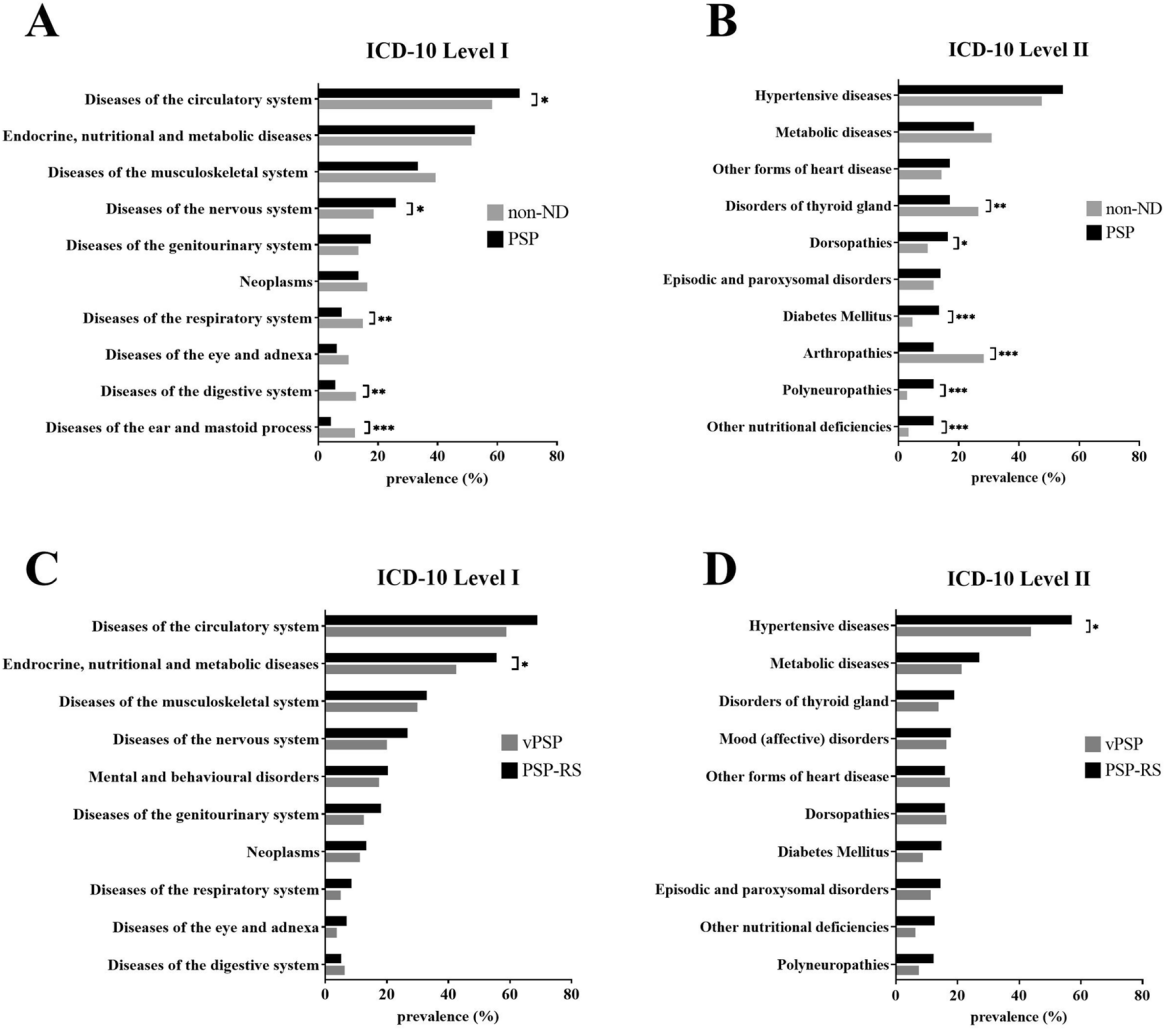
Prevalence of comorbidities according to ICD-10 classification. *p < 0.05, **p < 0.01, ***p < 0.001, Fisher’s exact test. Prevalence of the most common comorbidities on the first (**A**, **C**) and on the second level (**B**, **D**) of the ICD-10 classification. Comparison between PSP and non-ND patients (**A**, **B**) as well as in PSP-RS and vPSP (**C**, **D**). Abbreviations: *ICD* International Classification of Diseases, *vPSP* progressive supranuclear palsy-variants, *PSP-RS* progressive supranuclear palsy-richardson syndrome

Since neuropsychiatric disorders, in particular apathy and depression, are often part of the non-motor symptom complex of PSP, these disorders (F30-F39) were not included in the following comparison. The total number of comorbidities did not differ between PSP and non-ND patients (PSP, 3.4 ± 2.4; non-ND, 3.6 ± 2.3; *p* = 0.450). Two hundred and six (61.5%) PSP and 174 (63.3%) non-ND patients offered multimorbidity (Table 1). Figure 1A illustrates the prevalence of the ten most common comorbidities in PSP and non-ND patients corresponding to the chapters of the first level of ICD-10. PSP patients showed significantly more diseases of the circulatory system (PSP, 226 (67.5%); non-ND, 160 (58.2%); OR 1.49 [1.07–2.08]; *p* = 0.018) and the nervous system (PSP, 87 (26.0%); non-ND, 51 (18.5%); OR 1.54 [1.04–2.28]; *p* = 0.029) compared to non-ND patients. In particular, the prevalence of dorsopathies (PSP, 55 (16.4%); non-ND, 27 (9.8%); OR 1.80 [1.10–2.95]; *p* = 0.017), diabetes mellitus (PSP, 45 (13.4%); non-ND, 13 (4.7%); OR 3.13 [1.65–5.93]; *p* < 0.001), other nutritional deficiencies (PSP, 39 (11.6%); non-ND, 9 (3.3%); OR 3.89 [1.85–8.19]; *p* < 0.001) and polyneuropathies/other disorders of the peripheral nervous system (PSP, 39 (11.6%); non-ND, 8 (2.9%); OR 4.40 [2.02–9.58]; *p* < 0.001) was significantly higher in PSP patients according to the second level of ICD-10 (Fig. 1B). In contrast, non-ND patients showed more diseases of the respiratory system (PSP, 26 (7.8%); non-ND, 41 (14.9%); OR 0.48 [0.29–0.81]; *p* = 0.005), diseases of the digestive system (PSP, 19 (5.7%); non-ND, 35 (12.7%); OR 0.41 [0.23–0.74]; *p* = 0.002) and diseases of the ear and mastoid process (PSP, 14 (4.2%); non-ND, 34 (12.4%); OR 0.31 [0.16–0.59]; *p* = 0.001) on the first level of ICD-10. The prevalence of arthropathies (PSP, 39 (11.6%); non-ND, 78 (28.4%); OR 0.33 [0.22–0.51]; *p* < 0.001) and disorders of the thyroid gland (PSP, 57 (17.0%); non-ND, 73 (26.5%); OR 0.57 [0.38–0.84]; *p* = 0.004) was higher in non-ND on the second level of ICD-10.

### Medication

**Fig. 2:**
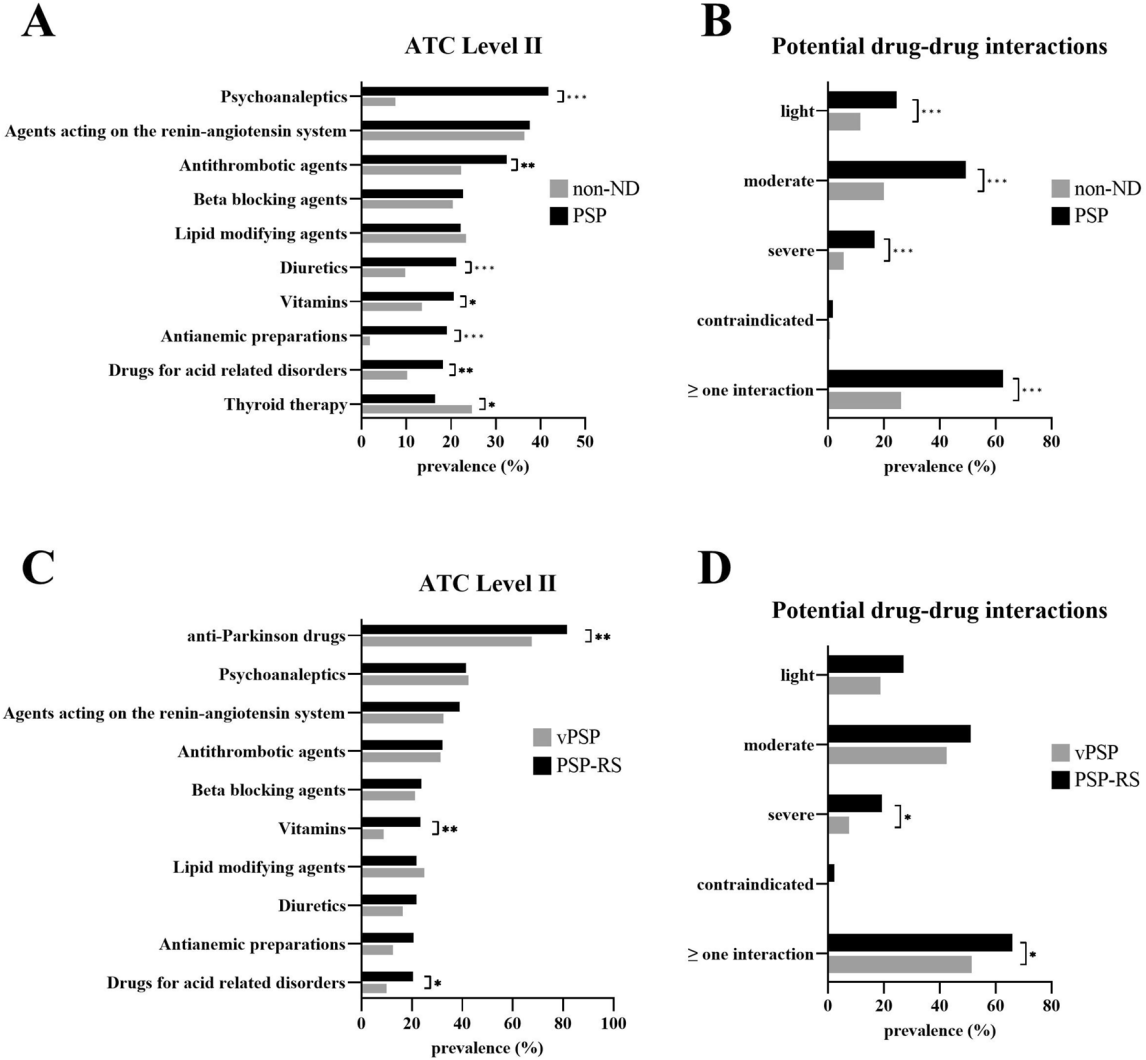
Prevalence of administered drugs according to ATC classification and potential drug–drug interactions. *p < 0.05, **p < 0.01, ***p < 0.001, Chi-squared test. Prevalence of the most common drugs administered on the third level of ATC system (**A**, **C**) and the prevalence of potential drug–drug interactions according to their severity (**B**, **D**). Comparison between PSP and non-ND patients (**A**, **B**) as well as in PSP-RS and vPSP (**C**, **D**). Abbreviations: *ATC* anatomical therapeutic chemical, *vPSP* progressive supranuclear palsy-variants, *PSP-RS* progressive supranuclear palsy-richardson syndrome

The medication was analyzed with the help of the WHO ATC classification. Both the number of patients with polypharmacy was significantly higher in PSP (PSP, 181 (54.0%); non-ND 56 (20.4%); OR 4.60 [3.20–6.61]; *p *< 0.001), and the number of administered drugs (PSP, 5.2 ± 3.0; non-ND, 2.8 ± 2.4; *p* < 0.001). This difference persisted even after exclusion of anti-Parkinson drugs (PSP, 4.1 ± 2.9; non-ND, 2.7 ± 2.4; *p* < 0.001). Figure 2A displays the ten most common administered drugs in PSP patients according to ATC Level II. In particular, the prevalence of psychoanaleptics (N06; PSP, 140 (41.8%); non-ND, 21 (7.6%); OR 8.68 [5.29–14.25]; *p* < 0.001), antithrombotic agents (B01; PSP, 109 (32.5%); non-ND, 61 (22.2%); OR 1.69 [1.18–2.44]; *p* = 0.005), diuretics (C03; PSP, 71 (21.2%); non-ND, 27 (9.8%); OR 2.47 [1.54–3.98]; *p* < 0.001), vitamins (A11; PSP, 69 (20.6%); non-ND, 37 (13.5%); OR 1.67 [1.08–2.58]; *p* = 0.021), antianemic preparations (B03; PSP, 64 (19.1%); non-ND, 5 (1.8%); OR 12.75 [5.05–32.18]; *p* < 0.001) and drugs for acid-related problems (A02; PSP, 61 (18.2%); non-ND, 28 (10.2%); OR 1.96 [1.22 – 3.17]; *p* = 0.005) was higher in PSP patients compared to non-ND. Thyroid preparations were administered more often in non-ND patients (H03; PSP, 55 (16.4%); non-ND, 68 (24.7%); OR 0.60 [0.40–0.89]; *p* = 0.011).

The CDSS AiD*Klinik*® was used to identify pDDIs. The data are shown in Fig. 2B. PSP patients exhibited significantly more pDDIs than non-ND patients (PSP, 1.4 ± 1.8; non-ND, 0.6 + 1.2; *p* < 0.001). Severe (PSP, 56 (16.7%); non-ND, 15 (5.5%); OR 3.48 [1.92–6.30]; *p* < 0.001), moderate (PSP, 165 (49.3%); non-ND, 55 (20.0%); OR 3.88 [2.70–5.59]; *p* < 0.001) and light interactions (PSP, 82 (24.5%); non-ND, 32 (11.6%); OR 2.46 [1.58–3.84]; *p* < 0.001) were significantly more frequent in PSP patients. The most common severe interactions in PSP patients were between diuretics/non-steroidal anti-inflammatory drugs (NSAIDs)/agents acting on the renin-angiotensin system (risk for acute kidney injury), acetylsalicylic acid/NSAIDs (attenuation of platelet aggregation inhibition), NSAIDs/selective serotonin reuptake inhibitors or selective serotonin-norepinephrine reuptake inhibitors (risk for gastrointestinal bleeding), central acting agents (e.g., melperone)/levodopa (increased or decreased effect of levodopa) and potassium-sparing agents/agents acting on renin-angiotensin system (risk for hyperkalemia). Amantadine, domperidone and amitriptyline were involved in most of the contraindicated combinations (risk of QTc-prolongation).

### Cardiovascular diseases

Based on the previous observations, aspects of the cardiovascular system were analyzed in detail. Diagnoses classified I05-09, I10-15, I20-25, I26-28, I60-69, I70-79 and Q20-28 according to ICD-10 level II were summarized as “cardiovascular diseases,” diagnoses classified G45 and I60-69 as “cerebrovascular diseases” and diagnoses classified E10-14, E78, I10-15 as “cardiovascular risk factors.” The prevalence of cardiovascular (PSP, 80 (23.9%); non-ND, 40 (14.5%); OR 1.84 [1.21–2.80]; *p* = 0.004) and cerebrovascular diseases (PSP, 39 (11.6%); non-ND, 13 (4.7%); OR 2.65 [1.38–5.06]; *p* = 0.002) was higher in PSP patients compared to non-ND. In addition to diabetes mellitus, the prevalence of ischemic strokes was higher in PSP patients (PSP, 32 (9.6%); non-ND, 9 (3.3%); OR 3.12 [1.46–6.56]; *p* = 0.002). Looking at the subgroup of diabetics, the number of untreated patients was larger in PSP patients (PSP, 25/45 (55.6%); non-ND 1/13 (7.7%); OR 15.00 [1.80–125.35]; *p* = 0.003), whereas the groups of treated or insulin-dependent diabetics did not differ. Unlike antithrombotic drugs (s. 3.4.), the number of cardiovascular drugs (ATC C) and antidiabetics (ATC A10) did not significantly differ between PSP and non-ND patients.

### Comparison of PSP subgroups

For the PSP subgroup comparisons, all collected PSP patients from the ProPSP and DescribePSP study were included without any selection (*n* = 350). This PSP cohort was classified into PSP-RS (*n* = 270, 77.1%) and vPSP phenotypes (*n* = 80, 22.9%) using the MDS-diagnostic criteria and MAX rules (Figs. 1, 2) [17]. Three hundred and eighteen PSP patients were diagnosed with a certainty of “probable” (90.9%), 15 with “possible” (4.3%) and 17 with “suggestive of” (4.8%) according to the current MDS-PSP-criteria [16]. Table 2 shows some clinical characteristics of all PSP patients and the subgroups PSP-RS and vPSP. Patients with vPSP phenotypes were significantly less likely to be female (PSP-RS, 133 (49.3%); vPSP, 27 (33.8%); *p* = 0.014). Most of the collected clinical scores indicate a higher disease burden for patients with PSP-RS (Table 2).

**Table 2:** Disease-specific characteristics and anti-Parkinson drugs (ATC N04) in PSP patients

	PSP (*n* = 350)	PSP-RS (*n* = 270)	PSP-Variants (*n* = 80)
Age, mean ± SD (min, max)	70.4 ± 7.4 (51, 88)	70.7 ± 7.2 (51, 88)	69.2 ± 7.9 (52, 86)
Sex, female (%)	160 (45.7)	133 (49.3)	27 (33.8)*
Diseases duration, mean ± SD, years *n* = 349	4.1 ± 3.0	4.2 ± 3.0	3.7 ± 2.8
CGI, mean ± SD *n* = 325	4.4 ± 1.2	5.5 ± 1.2	4.0 ± 1.3***
MoCA, mean ± SD *n* = 282	21.0 ± 6.0	21.0 ± 5.8	21.0 ± 6.6
PSPSS, mean ± SD *n* = 328	3.3 ± 1.0	3.5 ± 0.9	2.5 ± 1.0***
PSP-RS, mean ± SD n = 329	38.2 ± 15.7	41.1 ± 14.5	28.7 ± 15.8***
GDS-15, mean ± SD *n* = 293	6.1 ± 4.2	6.3 ± 4.1	5.6 ± 4.6
LED total, mean ± SD, mg	418.4 ± 360.6	435.5 ± 356.2	360.9 ± 371.9*
Levodopa, *n* (%)	237 (67.7)	190 (70.4)	47 (58.8)
Dopamine agonists, *n* (%)	30 (8.6)	25 (9.3)	5 (6.3)
MAO-inhibitors, *n* (%)	19 (5.4)	14 (5.2)	5 (6.3)
COMT-inhibitors, *n* (%)	9 (2.6)	7 (2.6)	2 (2.5)
Amantadine, *n* (%)	81 (23.4)	71 (26.3)	11 (13.8)*
Anticholinergics, *n* (%)	2 (0.6)	2 (0.7)	0 (0)

Abbreviations: *ATC* anatomical therapeutic chemical; *PSP* progressive supranuclear palsy; *PSP-RS* progressive supranuclear palsy-richardson syndrome; *LED* levodopa equivalent dose; *MAO* monoamine oxidase; *COMT* catechol-O-methyltransferase; *SD* standard deviation; *CGI* clinical global impression; *MoCA* montreal cognitive assessment; *PSPSS* progressive supranuclear palsy staging system; *PSP-RS* progressive supranuclear palsy rating scale; *GDS-15* geriatric depression scale-15

^*^p < 0.05, ***p < 0.001, Mann–Whitney *U* test or Chi-squared test

While the number of comorbidities between the PSP subgroups differed (PSP-RS, 3.67 ± 2.40; vPSP, 3.10 ± 2.44; *p* = 0.04), the pattern according to the first and the second level of ICD-10 was comparable (Fig. 1C, D). Only endocrine, nutritional and metabolic diseases on the first level (PSP-RS, 150 (55.6%); vPSP, 34 (42.5%); OR 1.69 [1.02–2.80]; *p* = 0.040) and hypertensive diseases on the second level of ICD-10 (PSP-RS, 154 (57.0%); vPSP, 35 (43.8%); OR 1.71 [1.03–2.82]; *p* = 0.036) were significantly more prevalent in patients with PSP-RS.

More anti-Parkinson drugs (PSP-RS, 1.14 ± 0.79; vPSP, 0.88 ± 0.77; *p* = 0.006) in a higher LED (PSP-RS, 435.48 ± 356.15 mg; vPSP, 360.93 ± 371.90 mg; *p* = 0.049) were administered to patients with PSP-RS. In particular, patients with PSP-RS took amantadine more frequently (PSP-RS, 71 (26.3%); vPSP, 11 (13.8%); OR 2.23 [1.12–4.47]; *p* = 0.020) in a higher LED (PSP-RS, 57.59 ± 111.46; vPSP, 30.63 ± 91.23; *p* = 0.023).

Except from vitamins (PSP-RS, 63 (23.3%); vPSP, 7 (8.8%); OR 3.17 [1.39–7.24]; *p* = 0.004) and drugs for gastro-esophageal reflux disease (PSP-RS, 55 (20.4%); vPSP, 8 (10.0%); OR 2.30 [1.05–5.06]; *p* = 0.034), the number of the other commonly administered drugs according to ATC level II was comparable between PSP-RS and vPSP (Fig. 2C). The mean number of pDDIs differed significantly between the PSP subgroups (PSP-RS, 1.54 ± 2.00; vPSP, 0.99 ± 1.35; *p* = 0.01), especially severe interactions were more frequent in patients with PSP-RS (PSP-RS, 52 (19.3%); PSP vPSP, 6 (7.5%); OR 2.94 [1.21–7.13]; *p* = 0.016; Fig. 2D).

## Discussion

To our knowledge, this is the first systematic analysis of common comorbidities and relevant aspects of drug therapy in a large cohort of PSP patients compared to a multicenter cohort of patients without neurodegenerative diseases. PSP patients presented a specific profile of comorbidities, especially a considerably higher prevalence of cardiovascular and neurological diagnoses. In particular, diabetes mellitus, cerebrovascular diseases and polyneuropathies were found more frequently in PSP patients. Hence, more antithrombotic drugs and antidepressants were prescribed to PSP patients, but not cardiac or antidiabetic drugs.

So far, only few studies investigated the prevalence of cardiovascular diseases in PSP patients. The most reliable data in this regard are available for arterial hypertension. In two large cohorts of PSP patients from Germany and North America, the prevalence of arterial hypertension was 48% and 57%, respectively [4, 5]. Another study detected arterial hypertension in 50% of autopsy-confirmed PSP cases [23]. These data reflect the prevalence of hypertension in our cohort (54.6%). Moreover, diabetes mellitus was more prevalent in our PSP cohort compared to non-ND patients. The current literature indicates a prevalence of approximately 15% in the age of 70–79, which is comparable to that of the PSP group (13.4%) [24]. Lastly, PSP patients showed a significantly higher prevalence of cerebrovascular diseases than non-ND patients. With approximately 9.6%, the demonstrated prevalence in PSP was comparable to the age-matched prevalence of ischemic stroke in Germany, but considerably lower than in PSP patients prior to diagnosis [3, 25]. However, the observed differences in the prevalence of cardiovascular diseases and diabetes between PSP and non-ND patients could be based on a lower prevalence of these diseases in the non-ND group compared to data from other Western countries [24–26].

An association between cardiovascular diseases, diabetes and neurodegenerative diseases is broadly assumed [27, 28]. A recent review attempted to illustrate the role of certain risk factors in Parkinson’s disease (PD) and cardiovascular diseases [28]. Based on common factors that increase (diabetes mellitus, male sex) or decrease (physical activity, moderate coffee consumption, female sex) the risk for both PD and cardiovascular diseases, the authors hypothesized shared pathophysiological pathways involving metabolic and inflammatory processes [28]. Previous reports have also shown an association between cerebrovascular and certain neurodegenerative diseases [29, 30]. Beside neurodegenerative diseases directly caused by a stroke, atherosclerosis and small-vessel disease was frequently detected as copathology in PD, Alzheimer’s disease (AD) and even in PSP.[31–33] Moreover, the emerging basic scientific and epidemiological evidence suggest a linkage between diabetes mellitus and neurodegenerative diseases [27, 34–36]. A recent meta-analysis showed that patients with diabetes were not only at higher risk for developing PD, but disease progression was also accelerated [35, 37]. In addition, Uyar et al. demonstrated poorer cognitive functioning in patients with PD and comorbid diabetes [34]. In this group of PD patients with cognitive decline, higher levels of serum neurofilament light chain (NfL) were detected, indicative for increased neuronal damage. The latter results are consistent with previous reports [38, 39]. Kwasny et al. analyzed pre-diagnostic features of a subsequent PSP diagnose in general practice and demonstrated for the first time an association between diabetes mellitus and PSP.[3] Interestingly, the group of untreated diabetics was markedly larger in the analyzed PSP cohort (25/45, 55.6%) compared to the non-ND group (1/13, 7.7%). This difference could be overestimated due to the small number of diabetics in the non-ND group. On the other hand, possible preventive effects of antidiabetic drugs could be considered. A number of epidemiological studies have examined the effect of antidiabetic drugs on AD, but obtained controversial results [27, 40, 41].

However, whether there is an indirect association via a common predisposition or a direct causal relationship of cardiovascular diseases, diabetes and neurodegenerative diseases remains a subject of much debate. A possible causality between cardiovascular diseases, diabetes and tauopathies is best described for AD. Baglietto-Vargas and colleagues extensively discussed the impact of diabetes on various pathophysiological processes involved in AD [27]. The disease-specific hyperglycemia and insulin resistance could initiate signaling pathways that impair neuronal glucose metabolism and thus stimulate phosphorylation and cleavage of tau as a cornerstone of tau accumulation and tau-mediated neurodegeneration [42, 43]. Furthermore, the accumulation of tau and β-amyloid in AD can be accelerated in the context of cardio- and cerebrovascular diseases [44, 45]. Due to a reduced cerebral blood flow and resulting hypoxia-induced ischemia, cerebrovascular diseases can induce a dysfunction of blood–brain barrier and mitochondria, enabling the deposition of misfolded proteins [44, 46]. On the other hand, cerebrovascular diseases and tau pathology appear to have a reverse association [47]. In this context, Kapasi and colleagues described increased tissue damage caused by small-vessel pathologies in the presence of β-amyloid and tau neurofibrillary tangles [48]. In addition to these causal considerations, the idea of a common predisposition or rather cause of cardiovascular diseases, diabetes and neurodegenerative diseases appears reasonable, since all of these result from an accumulation of misfolded proteins, for example β-amyloid or islet amyloid polypeptide (IAPP) [49, 50]. According to several hypotheses about the formation of misfolded proteins, molecular chaperones seem to play a crucial role [50]. Chaperones are highly conserved proteins that are an integral part of the proteostasis network regulation by acting as monitors for protein folding [51]. In the course of aging, various pathophysiological processes facilitate chaperone dysfunction and thus promote a disruption of proteostasis balance in favor of the accumulation of misfolded proteins [49, 52, 53]. More evidence is urgently needed to definitively answer these questions.

In our analysis, PSP patients suffered from significantly more pDDIs than non-ND patients. Since polypharmacy correlates directly with the number of pDDIs, the detected difference could be due to the higher number of administered drugs in PSP patients [54]. As previously described, the complex therapy of parkinsonism and associated comorbidities can facilitate polypharmacy [55, 56]. The prevalence of moderate and severe interactions in PSP patients was lower compared to a cohort of geriatric PD patients but considerably higher than reported in other cohorts of elderly [11, 55, 57]. Not only the sheer number of administered drugs, but also especially certain drugs, e.g., amantadine, amitriptyline and domperidone, pose a risk for pDDIs. The named drugs were involved in 66.7% of the contraindicated combinations in our PSP cohort because of their QTc-prolonging effects and consequent risk for cardiac arrhythmia [58–61]. Further, the most frequent severe interaction was between diuretics/NSAIDs/agents acting on the renin-angiotensin system. Known as “triple whammy,” this dangerous combination can cause acute kidney injury, especially at the start of treatment [62–64]. Since PSP patients show a non-negligible burden of cardiovascular diseases, which can often require the use of such a drug combination, and PSP patients represents a vulnerable group due to their disease-specific symptoms (e.g., dysphagia), pDDIs should be evaluated both at the beginning of a new drug therapy and during follow-up [65–67].

Admittedly, this systematic acquisition and analysis shows some limitations. Due to diseases-specific symptoms, such as early cognitive dysfunction, the collection of a detailed medical history can be prolonged, incomplete or not possible from patients themselves [16]. Therefore, the interviewer is sometimes dependent on questioning caregivers which may lead to loss of information but avoids anosognosia. This reporting bias between the groups was particularly noticeable for diseases long past or rather acute, for example appendicitis. Another level of this reporting bias presumably results from being diagnosed with a chronic neurological disease. Hereby, PSP patients regularly keep an appointment with a neurologist, which promotes the identification of new diagnoses (e.g., polyneuropathy). Another limitation concerns the reliability of the non-ND cohort. First of all, the non-ND group was only similar to the PSP patient in basic demographic parameters after selection for age. This could be caused by a selection bias, since usually patients without neurodegenerative diseases were selected for the non-ND cohort. These patients may tend to be generally healthier than other people in this age. However, this does not diminish the quality and validity of the data from PSP patients. In this way, these data provide important insights into prevalence of certain comorbidities in PSP patients with different phenotypes.

The magnitude and complexity of disease burden in the aging population is one of the major challenges in future medicine. The polypharmacy often used for drug treatment of elderly not only endangers the individual patient safety, but also places a burden on the healthcare system. For this reason, precise knowledge of typical comorbidities and pitfalls of drug therapy is crucial. In this study, we demonstrate for the first time the number and profile of comorbidities as well as key aspects of drug therapy in a large cohort of PSP patients. Due to the non-negligible number of comorbidities, in particular neurological and cardiovascular, a large proportion of PSP patients showed polypharmacy. The obtained insights can improve mindfulness and thus more drug safety in the treatment of PSP patients.

Moreover, the detected burden of cardio—and cerebrovascular diseases in PSP patients supports previous reports suggesting an association of cardiovascular diseases and neurodegenerative diseases. Further research may uncover the pathophysiological connection between the two disease spectra. Based on this, cardiovascular diseases could represent possible modifiable risk factors for the development of PSP.

## Data Availability

The data described in this manuscript were obtained from the DescribePSP, ProPSP and DANCER study. The data can be made available upon reasonable request. Requests to access the datasets should be directed to Stephan Greten, greten.stephan@mh-hannover.de.
